# Effectiveness of Psycho-Educational Intervention in HIV Patients’ Treatment

**DOI:** 10.3389/fpsyt.2014.00198

**Published:** 2015-01-15

**Authors:** Clarisse Ribeiro, Rui Sarmento e Castro, Mário Dinis-Ribeiro, Lia Fernandes

**Affiliations:** ^1^Hospital Joaquim Urbano (Centro Hospitalar do Porto, EPE), Porto, Portugal; ^2^Biostatistics and Medical Informatics Service and Centre for Research in Health Technologies and Information Systems (CINTESIS), Faculty of Medicine, University of Porto, Porto, Portugal; ^3^Research and Education Unit on Ageing (UNIFAI) and Centre for Research in Health Technologies and Information Systems (CINTESIS), Faculty of Medicine, University of Porto, Porto, Portugal

**Keywords:** medication adherence, empowerment, HIV, AIDS, psycho-educational program

## Abstract

Adherence to Highly Active Antiretroviral Therapy (HAART) is the main prognostic factor associated with HIV disease progression and death. The aim was to evaluate the effectiveness of a psycho-educational program to promote adherence to HAART in HIV patients. A longitudinal study (*n* = 102) over 9 months in an Infectious Diseases Hospital was carried out. Adherence to HAART was measured with standardized scales and values of viral load. Two groups were defined: adherents and non-adherents. In the latter, a psycho-educational program was implemented and 6 months later measured adherence to HAART. Knowledge about the infection, CD4 T lymphocytes and HIV-ribonucleic acid values were measured before and after this program. The sample was predominantly male (70%), heterosexual (78%), with a mean age of 49 (SD = 12.7) years, and 48% of participants were not adhering to HAART. After the program, non-adherence decreased to 21.6%. Knowledge about the infection increased from 79 to 97%. A significant increase in CD4 T lymphocytes (mean 540–580) and a decrease in viral load (mean 5411–3052) were observed, the latter of statistical significance. This program seems to be feasible and efficient, improving adherence to HAART.

## Introduction

HIV infection represents a significant cause of illness and death worldwide (1). The patterns of infection and associated diseases are continuously developing, with the emergence of new cases occurring throughout the world. The main epidemiological feature is the prevalence and incidence of infection, varying from place to place, as a consequence of numerous factors such as lifestyle and behavior, social and economic development, accessibility to health care, and the local epidemiological pattern (1, 2). Therefore, the treatment must be contextualized in a holistic view of the individual, family, and community and must be understood as a priority (1, 3, 4). Although a cure is not yet available, there are drugs, which are highly effective in controlling the disease, the Highly Active Antiretroviral Therapy (HAART). HAART has greatly contributed to improved survival and decreased morbidity and mortality among individuals infected with HIV/AIDS (5–9). In fact, HAART can reduce the rate of progression to AIDS or death in 86% of cases (10). However, failure rates can occur, because HAART reveals a wide variability in plasma concentrations achieved (7). This variability may be due to several factors including absorption, metabolism, clearance, drug interactions, and the adherence to therapy (7, 11, 12). From this perspective, it will be difficult to accurately predict the actual concentration that individuals will achieve. The effectiveness of HAART is now unquestionable, but there are also known adverse effects and toxicity that can manifest in the short or long term and that sometimes prevent the patient from adhering strictly to the therapeutic regime (12–17). Inadequate antiretroviral drug adherence leads to sub-therapeutic levels providing selective pressure and consequently adding pharmacological resistance (18–21). The main prognostic factor associated with disease progression and death is non-therapeutic adherence, requiring compliance with 95% of medication to achieve viral suppression and a stable preservation of immune function (22–24). Its absence leads to public health problems, since in addition to the lack of response to treatment, resistance to antiretroviral treatment will emerge quickly, which not only jeopardizes the future treatment regimens for the patient, but also for others, due to contamination by strains already resistant to drugs (25–27). Non-adherence is multifactorial and differs between patients. Different studies show different factors that influence therapeutic adherence. Socio-demographic factors are not associated with adherence (28, 29). However, psychological factors such as motivation, depression, and factors related to social support, support of health services, and information reveal a strong association with adherence to therapy (30–39). The increased life expectancy achieved as a result of HAART may have an impact on psychosocial and psychopathological reactions. The emotional reaction to infection can assume different levels and can change during the evolution of the disease (34). Nowadays, the patient no longer lives with the certainty of premature death, but still feels the stigma of HIV infection (38, 40–42). Often the sense of life being under threat is replaced by a continuing reassessment of its meaning (43). Recent studies show that HIV patients suffer a significant degree of psychological distress, manifested by a high prevalence of anxiety and depression (varying between 22 and 34%, and 26 and 41%, respectively) (42, 44–46). In terms of serious psychological symptoms, the literature shows a high prevalence of suicidal ideation (47). There are still quite frequent psychotic disorders in the advanced stages of the disease, and disruptions arising from the use of drugs or alcohol (48). Although patients now have new hope, stress, and emotional pain become constant and consequently affect their ability to rebalance and reintegrate their lives (49, 50). HIV infection may lead the individual to the destruction of their life goals, as well as absence of self-control and autonomy. The feelings of loss and grief and a sense of their new responsibilities are major stressors and threats to psychological well-being (49, 50). This infection is the cause of enormous suffering and socio-economic consequences. Therefore, strategies of psychological intervention to support therapeutic adherence become essential, since the first treatment regimen is considered the best opportunity for long-term control of viral replication (1, 21, 30, 51, 52). The commitment of all health professionals in the promotion of adherence to antiretroviral therapy is essential, using innovative intervention strategies and involving the patient and family in their own treatment decisions (1, 21). One strategy may be the implementation of interventions based on psycho-educational programs (30, 53–56). In one of the studies (30), the authors compared two interventions to increase HIV adherence, one based on cognitive-behavioral, motivational interviewing, and problem-solving techniques, and the other one on self-monitoring strategies, such as pill-diaries. Both interventions led to improvements in adherence at follow-up, with the first one showing more rapid results. Balfour et al. (54) conducted a randomized controlled trial with a psycho-educational program, verifying that this intervention not only reduced depressive symptomatology, but also enhanced HIV treatment by providing the preparation for HAART.

Considering these prior results, the purpose of this study was to evaluate the effectiveness of psycho-educational intervention conducted in a Portuguese hospital specializing in infectious diseases, in promoting adherence to HAART of HIV patients who were not drug addicts.

## Materials and Methods

### Participants

**Figure 1:**
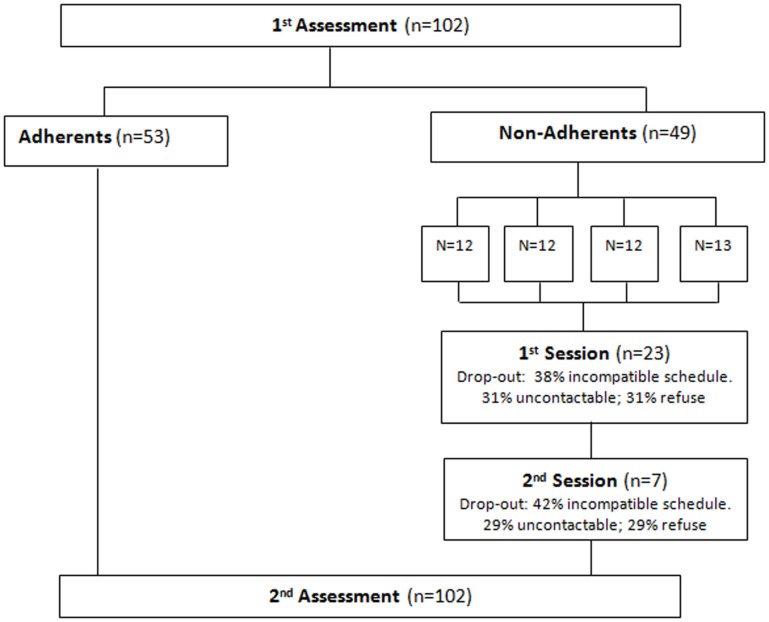
**Flow chart**.

A sample of consecutive patients (*n* = 102) was selected in a central hospital specializing in treating HIV patients in Portugal during 3 months (Figure 1). The use of illicit drugs was established as an exclusion criterion, since patients with a history of substance abuse already follow a specific program in this hospital, and as inclusion criteria, to be able to answer the questionnaires and have a basic level of education. Each patient received detailed information about the study and after that their informed consent was given and approval of the regional research ethics committee was obtained. Socio-demographic and family characteristics, health services use, and knowledge about the disease were assessed in a structured interview. Clinical data were also collected, including medication use, HIV-ribonucleic acid (HIV-RNA), and CD4 T lymphocytes values.

### Measures

#### Perceived satisfaction with social support

The perceived satisfaction with social support was assessed with the Scale of Satisfaction with Social Support (SSSS) (57). This scale aims to assess satisfaction with perceived social support in adults. It is a self-assessment scale with 15 items, presented as a set of statements, which are spread over four distinct domains, namely “satisfaction with friends,” “intimacy,” “satisfaction with family,” and “social activities.” The individuals must indicate the extent to which they agree with each statement on a scale of five ordinal positions, which are: “strongly agree,” “agree mostly,” “neither agree nor disagree,” “mostly disagree,” and “disagree totally.” The “satisfaction with friends” domain evaluates satisfaction with friendships and includes five items; the “intimacy” domain assesses the perception of the existence of intimate social support and includes four items; the “family satisfaction” domain assesses satisfaction with family support and includes three items; and the “social activities” domain assesses satisfaction with social activities and includes three items.

In the original validation study (57), the principal components analysis revealed the presence of four components, which explain 63.1% of the total variance. Globally, this instrument revealed good psychometric properties, regarding internal consistency (α = 0.85 for total, α = 0.64–0.83 for components), concurrent and discriminant validity.

#### Anxiety and depression

The Hospital Anxiety and Depression Scale (HADS) (58, 59), was designed by Snaith and Zigmond and it is used as a support to clinicians for identification and recognition of emotional components associated with physical illness. It is a scale that has been shown to be useful in hospitals, primary health care, and psychiatric settings and is considered a good instrument for screening anxiety and depression. It consists of two sub-scales, each one with seven items, measuring anxiety and depression. Both sub-scales are scored separately. The individual answers each item in an ordinal scale of four positions (0–3). The scores for each sub-scale range from 0 to 21. Scores are considered as follows: “normal” between 0 and 7, “mild” between 8 and 10, “moderate” between 11 and 14, and “severe” between 15 and 21. In the present study, the Portuguese version validated by Ribeiro et al. (60) was used. The Portuguese version revealed good psychometric proprieties, similar to the original one, as well as to the other adapted versions in other languages.

#### CD4 T lymphocytes

A CD4 T lymphocytes count was performed by collecting blood samples before and after implementation of the intervention program. The samples were collected by nurses at the hospital where the study occurred, using sterile vacuum tubes. The samples were analyzed in a specialized laboratory (Instituto Ricardo Jorge, in Lisbon) and the results were validated by a clinical hematologist.

#### HIV-ribonucleic acid

Levels of HIV-RNA in the blood were measured before and after implementation of the intervention program by amplification of viral RNA in plasma and by reverse transcriptase polymerase chain reaction (PCR) method in real time. Analyses were performed at the Instituto Ricardo Jorge in Lisbon. It was considered in this study that the patient had suppressed viral load if HIV-RNA values in the blood were less than 50 copies.

#### Patient’s knowledge related to HIV infection

A multiple-choice questionnaire was developed by the research team to assess the knowledge that patients had in relation to HIV infection. The questionnaire assesses issues like transmission, prevention, and treatment and was administered at baseline and after the psycho-educational intervention.

#### Adherence to HAART

Adherence to HAART was measured through the simplified Scale for Detecting Problems of Adherence to Antiretroviral Therapy (SDPAAT) (61). This is a dichotomous scale and comprises six items. Each item with a positive response scored one point and a negative response zero points. The total score is the sum of the items (minimum one and maximum six points). The first two questions of the scale must be positively answered in order to consider the patient free of problems with adherence. If both are negative, the degree of adherence is one, independent of the rest of the scores. It is considered that a patient has no adherence problem if they are rated between 5 and 6 points.

In the original validation study, the scale showed good psychometric proprieties, namely related to sensitivity 0.93 (CI95% 0.78–0.99), specificity 0.70 (CI95% 0.51–0.84), and a likelihood ratio of 3.08 (CI95% 1.28–7.39). The validation process for the Portuguese population includes its administration to the sample of the present study, after its independent translation and back-translation process by bilingual Spanish–Portuguese specialists.

### Procedures

The different measures were completed by patients. The main outcomes considered were adherence to HAART, proportion of incorrect answers about HIV infection, and values of CD4 and HIV-RNA. These outcomes were reassessed 6 months after the first intervention.

Patients classified as non-adherent to HAART (*n* = 49) were submitted to a group intervention program, comprising two sessions, sponsored by a specialist community health nurse. Each patient was called by phone for each session. The sample of non-adherents was divided into three groups with 12 members each and one group with 13. All of them were questioned about their availability at the time of contact. In the case of no response to the first phone call, three further calls were made on different days. Each session lasted 120 min. The sessions were developed in the training room of the hospital on weekdays (Monday to Saturday), alternating between the morning and afternoon in order to maximize the likelihood of patient attendance.

#### Implementation of the intervention program

The program was implemented by a specialist community health nurse, experienced in community intervention programs, and was based on a project methodology (62, 63). The two sessions were implemented following a manual developed by the research team, with objectives, subjects, strategies, and resources, based on Health Education Action for groups with HIV infection and antiretroviral therapy. An expository methodology with a theoretical and interactional program, using multimedia (Powerpoint) was chosen. In the first session, issues related to the pathogenesis of infection, prevention, and transmission were explained, as well as general principles of antiretroviral medication and the importance of adherence to therapy. During the presentation, the non-adherent sample patients were encouraged to participate with questions and suggestions. A second session consisted of an explanation about patients’ problems and feelings, and the promotion of shared difficulties and experiences as well as their empowerment, through discussion and problem solving. The topics were always diverse and chosen by the patients, consisting of the marginalization and prejudice that they often feel and the fear of colleagues and neighbors on discovering their illness. Legal issues about bank loans were discussed, once they identified this as an important problem. Strategies were highlighted to minimize forgetting to take medication and to discuss the importance of the availability of reading material about HIV/AIDS in the general population, in order to destigmatise the disease. During the sessions, the promotion of a calm and cozy environment was found to be important to develop a therapeutic relationship. At the end of each intervention a synthesis of the content of the discussion was carried out. During the group sessions there was no resistance to patients’ participation in the sessions due to the health problem in question. There was however a need to ensure the complicity of all participants, as it was a cohesive group, in which all were patients with HIV.

### Statistical analysis

The statistical analysis was performed with the Statistical Package for the Social Sciences – SPSS^®^ for Windows, version 17.0.

For the comparison of dependent or related groups, the parametric Student test for paired samples was used. When normality of the distributions could not be assumed, the Wilcoxon non-parametric test was used. Additionally, when appropriate, correlation analyses between variables of interest were performed with the Pearson correlation coefficient. The non-parametric chi-square test was also used to verify the associations between the baseline and final assessments and among other variables in the study. All analyses assumed 95% confidence interval (95% CI, two-tailed).

For the validation of SSSS and in order to extract common factors from the interpretation of the items relating to this scale, an exploratory factor analysis was performed (with varimax rotation) to explore the behavior of the scale with this particular population. Cronbach’s alpha was computed to assess the internal consistency of the validated scale and its factors.

The validation of SDPAAT was performed by calculating its sensitivity (ratio of non-adherents detected by SDPAAT and the true non-adherents) and specificity (ratio between adherents detected by SDPAAT and the true adherents).

Finally, to assess the significance of some variables on the likelihood of adherence to HAART, the logistic regression method Forward: LR (64–66) was used. Variables like gender, anxiety, depression, regular monitoring at the health center, answers to perceived needs, number of children, knowledge of the disease, admissions, and number of medication doses per day were explored.

## Results

### Socio-demographic characteristics

In the initial sample (*n* = 102), 70% of individuals were male and had an average age of 49 (SD = 12.7). Most were unmarried (56%) and almost all were Caucasian (97%). The majority were heterosexual (78%) and 70% had completed secondary education. The most common (35%) professions were in the tertiary sector (commerce, communications, health, and education).

### Family characteristics

In this study, 28% of patients lived alone. Approximately 65% had children and 76% considered they spent enough time with their family. Among the patients, 16% believed their health influenced their relationship with their family, and in 70%, the diagnosis of HIV was identified as the main event, which had disturbed the family relationship. The majority believed they needed family support (88%), and that their spouse or partner was the best person available to help.

### Clinical characteristics

Table 1 shows that the entire sample was infected with HIV-1, and the duration of infection was 7 years on average. About 28% of patients were classified (Centers for Disease Control and Prevention) as being in the AIDS stage and had a median of 344 CD4 T lymphocytes, and the majority had a viral load below 50 copies (78%). The majority of the patients were taking medications twice a day (67%), corresponding on average to four tablets per day. Most subjects referred had no side effects to HAART (81%). Only about 19% had side effects such as fatigue, insomnia, nausea, and pain. About 56% of the sample had already had to change treatment, the main reasons being metabolic changes (50%) and resistance to antiretroviral drugs (25%). Almost 30% had been hospitalized between one and three times.

**Table 1:** **Clinical characteristics of sample**.

(*n* = 102)	(%)
HIV-1	100
CDC: phase of AIDS	28
Time of infection ≥5 years	72
CD4 T lymphocytes
<200	8
201–350	28
>351	64
Viral load
≤50	78
>50	22
Tablets per day
Mean	3.8
Min./Max	1/11
Without ARV side effects	81
Hospitalized due to HIV	27
Anxiety (HADS)
Absent	47.1
Mild	26.5
Moderate	13.7
Severe	12.7
Depression (HADS)
Absent	71.6
Mild	15.7
Moderate	9.8
Severe	2.9

*^a^ Multiple-choice questions (number of valid cases, % of valid cases and total valid cases)*.

According to HADS, it was verified that 52.9% suffered from anxiety, ranging in level from mild to severe (26.5% mild, 13.7% moderate, and 12.7% severe). In relation to depression, 28.4% scored for depression (15.7% mild, 9.8% moderate, and 2.9% severe).

Through analysis of Table 2 it was concluded that according to the SSSS, “Satisfaction with family” had a mean of 1.8 (SD = 1.2), “Satisfaction with friendships” a mean = 2.1 (SD = 1.2), “Intimacy” a mean = 2.4 (SD = 1.3), and “Social Activities” a mean = 3.3 (SD = 1.2).

**Table 2:** **Satisfaction with social support (SSSS) – 1^st^ assessment**.

(*n* = 101)	Mean (±SD)	No. items	Min.	Max.
Satisfaction with family	1.85 (± 1.16)	3	1	5
Satisfaction with friendships	2.09 (± 1.22)	4	1	5
Intimacy	2.37 (± 1.26)	2	1	5
Social activities	3.33 (± 1.21)	6	1	5

Regarding the SSSS factorial analysis, revealed the presence of four components explaining 68.6% of the total variance in this sample of HIV patients, namely Social Activities, which explained 35.1%, Satisfaction with friendships (14.6%), Satisfaction with family (11.9%), and Intimacy (7.1%). All the factors found showed good internal consistency (α = 0.83, 0.87, and 0.88, respectively), except for Intimacy (α = 0.42), and all significantly correlated (*p* < 0.01) with the SSSS total score.

The present study is part of the validation process of the Portuguese version of SDPAAT, tested in the total sample of 102 patients. The scale showed a sensitivity of 0.61 (CI 95% 0.52–0.71) and a specificity of 0.82 (CI 95% 0.74–0.89), when compared with viral load values, used as gold-standard. Using the SDPAAT scale, 48% (*n* = 49) of the total sample (*n* = 102) was classified as non-adherent. In relation to SSSS dimensions and HADS sub-scales, no significant differences were found in the mean values between the group of adherent and non-adherent, classified by SDPAAT.

### Interventional program

In the interventional program, it was found that 43% of non-adherents to HAART participated in the first session, and 14.3% in the second session. Of the non-adherents to HAART, 69 and 71% reported that their absence in the first and second sessions, respectively, was due to work commitments or contact difficulties.

Once the intervention program had been implemented, a significant increase in the values of CD 4 lymphocytes was found (*p* = 0.001) between the first (mean = 540.0, SD = 354.0) and final moment (mean = 580.1, SD = 328.3) (Table 3).

**Table 3:** **Comparison of CD 4 T lymphocytes and viral load before and after the program**.

	*N*	Mean (±SD)	*t*	*p*-Value^a^
**(*n* = 102) CD 4**				
Initial/M0	102	540.02 (±353.99)	−3.382	0.001
Final/M9	102	580.15 (±328.34)	
**(*n* = 102) VIRAL LOAD**				
Initial/M0	102	5.410.73 (±32 011.36)	0.663	0.509
Final/M9	102	3.052.31 (±18 191.71)	

*^a^ Results according to the Student’s *t*-test for two paired samples, with 95% confidence*.

In relation to the viral load outcome, despite the average reduction before and after the implementation of the intervention program, this difference was not statistically significant (*p* = 0.509) (Table 3).

In both groups, the CD4 lymphocytes significantly increased between the first and second assessments (adherents, from 538 to 581, *p* < 0.01; non-adherents, from 360 to 416, *p* < 0.05).

Analyzing the data of Table 4, a significant association between adherence before and after implementation of the intervention program was observed (*p* = 0.002). In fact, most of the individuals who had initially adhered to therapy remained until the end (91%). After the implementation of the psycho-educational program, 65% of the initial non-adherent individuals became adherents to HAART. In all samples, the adherence to HAART after the implementation of the program increased from 52 to 78%.

**Table 4:** **Comparison of adherence to HAART between groups (adherents and non-adherents), at 1^st^ and 2^nd^ assessment**.

		1^st^ Assessment (before the program)			
		Adherent	Non-adherent	Total	*p*-Value^a^
2^nd^ Assessment (after the program)	Adherent	48 (90.6%)	32 (65.3%)	80 (78.4%)	0.002
	Non-adherent	5 (9.4%)	17 (34%)	22 (21.6%)	
	Total	53 (100%)	49 (100%)	102 (100%)	

*^a^ According to the results of the chi-square with 95% confidence*.

In the present study, an additional analysis of the impact of some variables described in the literature as predictors of adherence to HAART was carried out. No variables were independently associated with the adherence to HAART, although gender (*p* = 0.054) and knowledge about infection (*p* = 0.051) almost reached statistical significance.

## Discussion

This study was the first intervention developed with Portuguese HIV patients, whose strategy for implementing the program consisted in a combination of behavioral and educational approaches, in a group intervention. The study suggests that the implementation of this psycho-educational program was effective in promoting adherence to HAART and leads to significant gains in the immune status of patients, especially in relation to CD4 T lymphocytes levels. These results are in line with the findings from previous studies [e.g., Ref. (30, 44, 45, 47, 48, 54)] that also concluded psycho-educational interventions could enhance adherence to HAART.

It was also observed in the present study that the implementation of the psycho-educational program significantly reduces the average HIV-RNA. Also a reduction in viral load values was observed, in spite of no statistical significance achieved. In relation to CD4 T lymphocytes, a statistically significant increase was observed.

Regarding adherence to HAART, it was found that the group of adherents continued to be adherents to HAART and the majority of the non-adherent individuals became adherents after the implementation of the psycho-educational program. Adherence to HAART was assessed using a scale based on the immune status of the patient, his attendance to collect his medication from the pharmacy and his knowledge regarding medication. This method was preferred over self-based assessment methods that could overestimate adherence, leading to imprecise estimates (47).

With the implementation of this program it was also found that patients’ knowledge about HIV infection, as assessed by the number of right answers on the questionnaire, improved. There was a significant decrease in the average number of wrong answers after the implementation of the intervention compared to baseline assessment.

It is important bearing in mind that viral suppression and immunological stability, and consequently the minor risk of opportunistic infections and malignancies depends on adherence to HAART. To remain adherent to treatment, the patient avoids or delays the potential resistance to HAART, thereby achieving a more extensive range of alternative therapies to delay disease progression. In that sense interventions with educational components about the disease and its treatment, are gaining relevance by showing effectiveness in improving adherence to medication.

Regarding the SSSS, the factor structure found in this study was not similar to that obtained in the original study (57), with three of the items regrouped in different factors. All the dimensions of the scale showed good internal consistency, achieving higher values than the original validation study, except for Intimacy. The extremely low value of Cronbach alpha related to the last dimension, could be explained by the small number of items.

Also in accordance with the original study (57), the SSSS revealed good discriminant validity, with statistically significant correlations between the total and domains as well as between items and domains.

The present study forms part of the validation process of SDPAAT. Compared with the original study (61), the Portuguese version of SDPAAT showed moderate sensitivity values and good specificity. However, these values must account for some methodological heterogeneity such as gold-standard considered (viral load values vs. pharmacy record about the medication delivered that was used in the original study).

Although some studies [e.g., Ref. (30, 38, 39)] presented psychological factors such as anxiety, depression and social support associated with adherence to therapy, emphasized the importance of exploring interventions to improve its diagnosis and treatment, in the present study no differences were found in these domains between adherent and non-adherent patients.

An aspect that may be relevant is the fact that before implementing the program contact between the research team and the patients was reduced. A continuing professional relationship that would allow the consolidation of trust and empathy was crucial for the aim of the program. These factors are essential when trying to develop such interventions. The rigorous study of the implementation and evaluation of intervention programs to promote adherence to HAART through psychological and educational methodology in a group is imperative, given that in clinical practice the resources are few, and it is crucial to its effectiveness. In this study, the methodology was developed using assessment scales validated for the Portuguese population (SSSS and HADS).

Some limitations of the present work resulted from the lack of a control group and the study’s experimental design.

Also it must be taken into consideration that these results may have been influenced by the additional contact *per se* with the health professional, when the assessments were performed. The literature shows that the rates of adherence to intervention programs continue at a lower level in spite of innovative programs (40, 67). In this study, it was observed that patients participated with interest in the sessions, above all in the first one. These patients participated actively in the discussions. However, lower adherence was found in the second session, perhaps because it coincided with the start of the school year. Some patients reported having work commitments. It is noteworthy that the majority of the study population was professionally active and some of them resided in a district outside the hospital area, which may explain their lack of availability to attend the sessions.

Further studies should be conducted with randomized clinical trials. It is essential that patients know the health professionals and that the co-construction of knowledge is based on empathy that is established between patient and professional. It is important that health professionals assume the commitment to foster targeted interventions with innovative models to promote care (40, 68–71). Developing intervention projects in this area is a continual challenge, especially for the health professionals involved. This study, due to its innovative nature, is undoubtedly an incentive to develop strategies for ongoing support, with efficiency in health promotion reflected in gains in the health of these populations.

## Author Contributions

Lia Fernandes and Mário Dinis Ribeiro contributed to the conception and design of the work. Clarisse Ribeiro participated in the data collection and in drafting the work, as well as in analysis and interpretation of data, with Rui Sarmento e Castro, Mário Dinis Ribeiro, and Lia Fernandes, who also critically revised the paper. All the authors have approved the final manuscript and agreed to be accountable for all aspects of the work in ensuring that questions related to the accuracy or integrity of any part of the work are appropriately investigated and resolved.

## Conflict of Interest Statement

The authors declare that the research was conducted in the absence of any commercial or financial relationships that could be construed as a potential conflict of interest.

## Supplementary Material

The Supplementary Material for this article can be found online at http://www.frontiersin.org/Journal/10.3389/fpsyt.2014.00198/abstract
