# Supplementary material for: Effectiveness of Psycho-Educational Intervention in HIV Patients’ Treatment
Source: Front Psychiatry. 2015 Jan 15;5:198. doi: 10.3389/fpsyt.2014.00198 (PMC4295437; doi:10.3389/fpsyt.2014.00198)
Supplement: Supplementary file 1 [file Data_Sheet_1.DOCX]

**Appendix**

Overview of the sessions of the psycho-educational program

Session 1: The first session of the program focuses on issues related to the pathogenesis of infection, prevention and transmission, general principles of antiretroviral medication and the importance of adherence to therapy.

**Pathogenesis of infection and symptoms:**

- Teach what HIV and AIDS mean and the differences between them
- Teach about HIV infection signs and symptoms, focusing on the fact that the infection cannot be seen in the face or body of the person (demystify the association of HIV infection and the poor condition of the person)
- Demystifying the stigma of HIV infection (associated with prostitution, addiction to drugs and homosexuality)

**Transmission:**

- Educate about transmission pathways, explaining that it is not transmitted by sharing spaces or objects such as silverware, dishes, etc
- Acknowledge that HIV infection is not transmitted in the care provided to children, grandchildren and other loved ones

**Prognosis of infection:**

- Clarify that HIV infection is now considered a chronic infection in which patients can have quality of life for many years, and is not a "death sentence"

**Treatment:**

- Clarify that HIV infection has no cure, but has a treatment that is highly effective
- Acknowledge that treatment is effective only if it is strictly accomplished
- Clarify that taking antiretroviral medication does not mean that you won’t go on with the infection
- Educate about possible side effects of antiretroviral therapy and how to minimize them (eg adjusting timings, if nausea or insomnia, etc.)
- Clarify that the side effects usually stop after some time
- Show the importance of contacting a healthcare professional (doctor or nurse) when necessary, never suspending or changing the prescribed treatment
- Educate about the need to continue with antiretroviral treatment for a lifetime, highlighting the fact that if you feel healthier it does not mean you can live without the treatment
- Promote the verbalization of barriers to antiretroviral therapy adherence
- Promote discussion about strategies to minimize the difficulties of therapy adherence verbalized

Session 2: The second session of the program is to discuss all issues that patients identified as essential (stigma, treatment, HIV vaccine, legal issues, privacy and confidentiality). Each patient brings his question or problem to the group discussion and the other patients give their opinion according to their own experiences.
